# Ulcerative Lesions: A Rare Cutaneous Manifestation of Brucellosis

**DOI:** 10.1155/2018/8643192

**Published:** 2018-05-16

**Authors:** Abbas Azadi, Payman Jafarpour Fard, Mohammad Almasian

**Affiliations:** ^1^Department of Infectious Diseases, Lorestan University of Medical Sciences, Lorestan, Iran; ^2^Student Research Committee, Lorestan University of Medical Sciences, Lorestan, Iran; ^3^Department of the English Language, School of Medicine, Lorestan University of Medical Sciences, Khorramabad, Iran

## Abstract

Brucellosis is a disease that is transmitted from animals to humans mainly via the consumption of unpasteurized dairy products, and it can involve any organ all over the body. Here, we report a significant rare case of brucellosis with cutaneous manifestations in a 52-year-old male patient whose disease was diagnosed via a serology test. The patient received standard antibiotic treatment, and his cutaneous lesions healed quickly. Although the cutaneous manifestations of brucellosis are exceedingly rare, in case of encountering ulcerative lesions and other cutaneous findings, particularly in endemic areas, infection with brucellosis should be kept in mind as an important differential diagnosis.

## 1. Introduction

Brucellosis is a communicable disease that can be passed between humans and animals [1]. Human infection is mostly observed among people who have to be in contact with diseased animals as a result of their jobs or among individuals who consume contaminated milk products or animal tissues. Very rare does this disease pass from person to person [2]. The complications and the social and economic consequences of this disease have made it a serious public health challenge [3]. Iran being known as an endemic area for brucellosis, the disease is highly prevalent in the Lorestan province [4].

As a systemic disease, brucellosis can affect any organ in the body [5]. However, the skin is not commonly affected by this disease, and cutaneous manifestations only occur in 1–14% of all cases [6, 7]. In this article, a rare case of brucellosis with cutaneous manifestations involving ulcerative lesions is described.

## 2. Case Presentation

**Figure 1:**
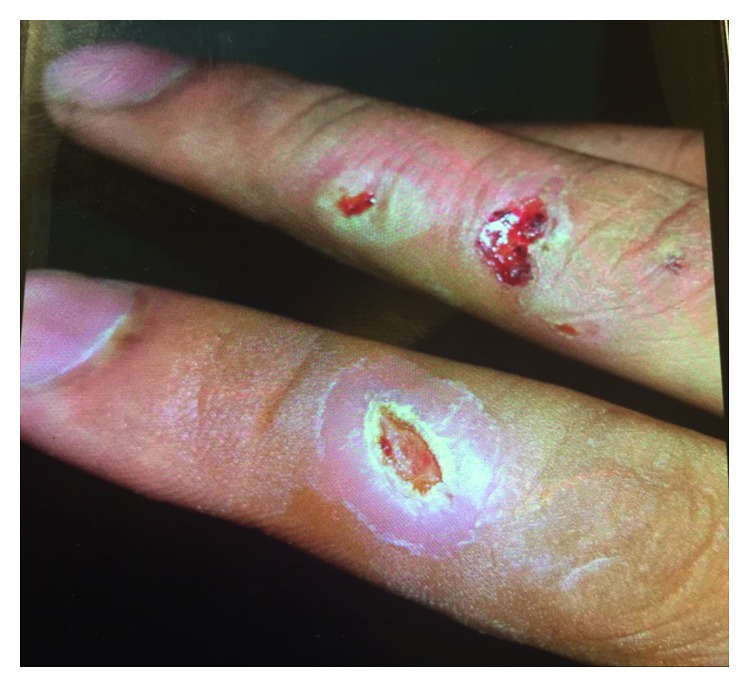
Ulcerative lesions before treatment.

A 52-year-old male patient referred to the infectious clinic of Shohadaye Ashayer Hospital in the Lorestan province of Iran. He complained about several ulcerative lesions on the back of his fingers (Figure 1). Both hands were involved. However, the ulcers were limited to the back of his fingers, not being observed on the palmar surface of his hands. The wrist, the forearm, and the rest of his upper limbs were not involved, either. The patient mentioned that his ulcers were not itchy, but they were painful. He did not complain about pain in his joints, but he had experienced fever and sweating, at times intense, particularly at nights. He stated that he had lost 5 kg of weight during the course of his disease. Neither the patient himself nor his family members had been affected by chronic conditions such as hypertension, diabetes, and so on. He had not taken any medications for a long period of time and was neither a smoker nor an addict. He said that he was a farmer.

On physical examination, he was conscious and his vital signs were stable. There was no organomegaly. Motor and sensory examination of the upper and lower limbs was normal. His pulse was normal. The only findings detected during physical examination were a high temperature and ulcerative lesions on his fingers.

The results of the laboratory tests were as follows: The hematology test showed that the white blood cell count was 9500/*µ*L (neutrophils 65%, lymphocytes 27%, monocytes 5%, and eosinophils 3%), the red blood cell count was 5.51 × 10^6^/*µ*L, the platelet count was 266 × 10^3^/*µ*L, Hb was 17 g/dL, hematocrit was 49%, MCV was 88 fL, ESR 1st hour was 5, and so on. The biochemistry test showed that urea was 26 mg/dl, creatinine was 0.9 mg/dl, calcium was 9.9 mg/dl, TSH was 1.0 ulU/ml, T3 was 0.8 ng/ml, and T4 was 8.9 *µ*g/dl. In the immunoassay tests, HIV and the hepatitis virus panel showed that HBsAg was negative and HIV Ag. Ab" I + II and HCV Ab were both nonreactive. The serology test showed that CRP was negative but the Wright agglutination test and 2ME (the Wright agglutination test is the first step in diagnosis of brucellosis, and the 2ME test is used as a complementary test) were both positive, being 1/60 and 1/80, respectively. Hereby, it was confirmed that ulcerative lesions on the fingers of the patient were due to infection with brucellosis. Therefore, standard antibiotic treatment for the patient was prescribed, and his lesions started to heal in a short period of time.

## 3. Discussion

As the most common zoonotic disease, brucellosis is an important infectious illness. Brucellosis is known as a serious challenge for the health system in the developing countries. Diverse bacterial species can cause brucellosis in humans, four of them being *Brucella melitensis*, *Brucella suis*, *Brucella abortus*, and *Brucella canis* [8]. In Iran, *B. melitensis* is the main reason for human infection [9].

According to reports by the WHO, more than 500,000 individuals are annually diagnosed with brucellosis throughout the world, especially in the developing countries [10]. The incidence of brucellosis is especially high in areas, like the Mediterranean region and the Middle East [11, 12]. Brucellosis is endemic in Iran, where the disease does not occur with the same frequency all over the country, the number of people affected by brucellosis varying between 98 and 130 per 10000 individuals. Brucellosis has the lowest incidence rate in the southern parts of Iran [13]. However, residents of central Iran and the Lorestan province are more likely to be infected with this disease [4].

Brucellosis is a systemic infection which can involve any organ or tissue in the human body. Cutaneous manifestations are only observed in approximately 5–10% of all cases of brucellosis [14]. Conditions such as direct inoculation, hypersensitivity, immune complex deposition, and also the direct invasion of the skin by bacteria via the hematogenous spread mechanism can lead to skin lesions [6, 7, 14, 15].

A study by Akcali et al. investigated the types and rates of cutaneous manifestations among brucellosis patients. They studied a total of 140 individuals who had already been diagnosed with brucellosis. Only 8 individuals (5.71%) with cutaneous presentations were found among the population, of whom there were 2 cases (25%) of maculopapular eruptions, 2 cases (25%) of erythema nodosum-like lesions, 1 case (12.5%) of psoriasiform lesions, 1 case (12.5%) of palmar erythema, 1 case (12.5%) of malar eruption, and 1 case (12.5%) of palmar eczema. Akcali et al. suggested that although skin lesions are not limited to brucellosis, the presence of such manifestations may indicate the incidence of brucellosis in an endemic area [16]. In another study, Vicaro et al. reported that a 29-year-old woman infected with *Brucella abortus* had developed maculonodular and purpuritic lesions [17]. Nagore et al. described the case of a 22-year-old man who had leukocytoclastic vasculitis as a cutaneous manifestation of brucellosis [18]. Chronic skin ulceration is one of the complications of brucellosis [19]. Balabanova-Stefanova et al. summarized and classified various cutaneous manifestations of brucellosis in a table in their review article, indicating that skin ulcers resulting from brucellosis are sporadic [20]. In the patient described in the present article, the cutaneous manifestations in the form of the ulcerative lesions were painful and had involved both hands due to occupational contact. As a result, it can be stated that this case represents a sporadic presentation of brucellosis in an endemic area of the Lorestan province.

**Figure 2:**
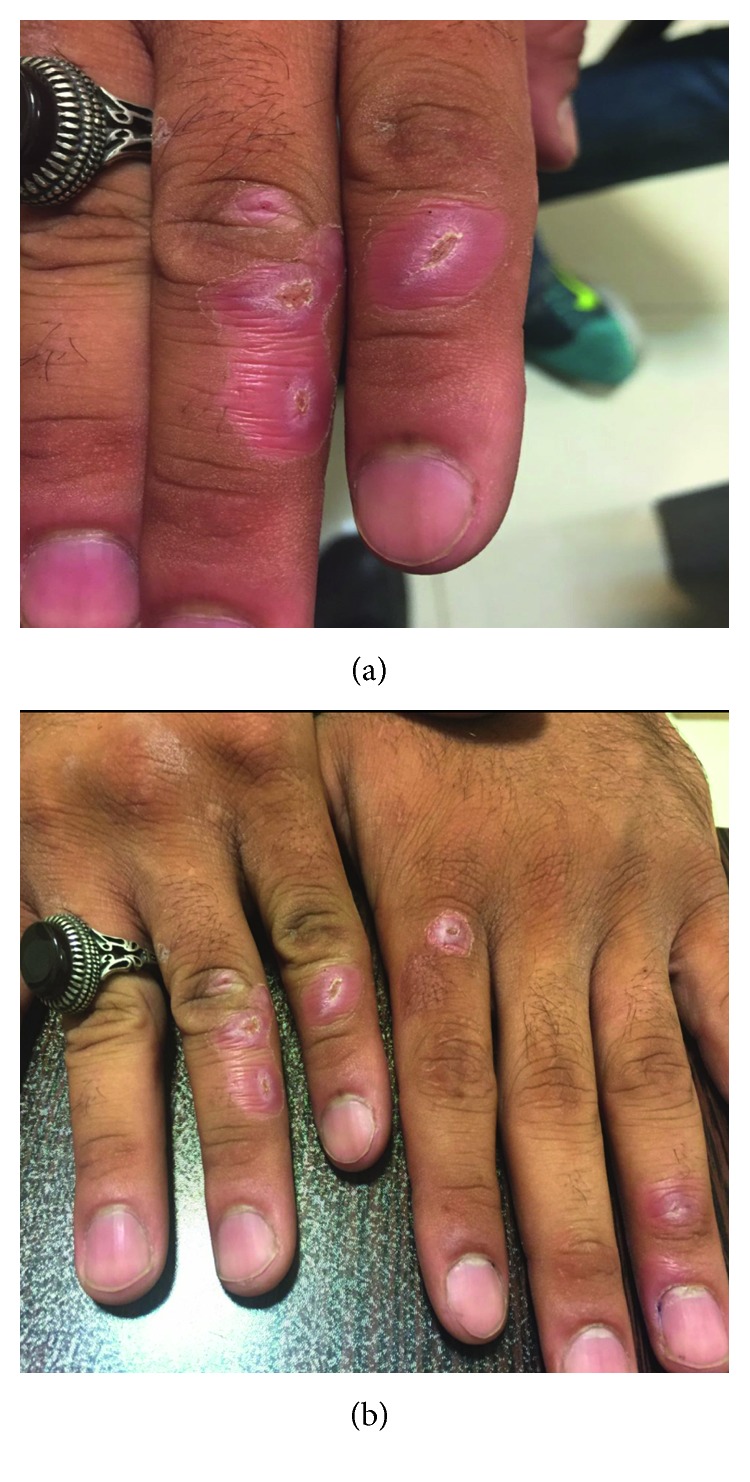
Ulcers healed 3 weeks after the start of the treatment.

The patient was a 52-year-old male farmer and rancher living in the Lorestan province, an endemic area for brucellosis with high brucellosis prevalence rates. In this province, people have a mostly traditional lifestyle, and the vast majority of the population live in villages and rural areas, where people are in close contact with cattle and livestock. On the other hand, animal products such as unpasteurized dairy products and contaminated meat are an important part of their daily diet. Therefore, people in this province and similar areas which are considered endemic for brucellosis are extremely susceptible to brucellosis. In summary, infection with brucellosis was confirmed via a positive serology test and the disproof of other differential diagnoses as well as an excellent response to standard antibrucellosis treatment in this case. The patient was treated on an outpatient basis, receiving standard antibrucellosis treatment, including streptomycin, doxycycline, and rifampin. Three weeks after the beginning of the standard antibrucellosis regimen, the patient's ulcerative lesions healed significantly (Figure 2). During the treatment course, the patient was followed up. No signs of relapse were observed, and full remission was ultimately achieved.

## 4. Conclusion

The cutaneous manifestations of brucellosis are noticeably rare and may overlap with dermatologic diseases. If a patient presents with painful and ulcerative lesions or other cutaneous manifestations, in areas, like the Lorestan province, where brucellosis is endemic, it is highly recommended that brucellosis should be taken into consideration as an important differential diagnosis.
